# Age-Dependent Invasion of Pseudorabies Virus into Porcine Central Nervous System via Maxillary Nerve

**DOI:** 10.3390/pathogens11020157

**Published:** 2022-01-26

**Authors:** Konstantinos Papageorgiou, Ioannis Grivas, Maria Chiotelli, Alexandros Theodoridis, Emmanuel Panteris, Dimitris Papadopoulos, Evanthia Petridou, Nikolaos Papaioannou, Hans Nauwynck, Spyridon K. Kritas

**Affiliations:** 1 Laboratory of Microbiology and Infectious Diseases, School of Veterinary Medicine, Aristotle University of Thessaloniki, 54124 Thessaloniki, Greece; dpapvet@hotmail.com (D.P.); epetri@vet.auth.gr (E.P.); skritas@vet.auth.gr (S.K.K.); 2 Laboratory of Anatomy, Histology and Embryology, School of Veterinary Medicine, Aristotle University of Thessaloniki, 54124 Thessaloniki, Greece; janos@vet.auth.gr (I.G.); mchiotel@vet.auth.gr (M.C.); 3 Laboratory of Animal Production Economics, Faculty of Health Sciences, School of Veterinary Medicine, Aristotle University of Thessaloniki, 54124 Thessaloniki, Greece; alextheod@vet.auth.gr; 4 Department of Botany, School of Biology, Aristotle University of Thessaloniki, 54124 Thessaloniki, Greece; epanter@bio.auth.gr; 5 Department of Pathology, Faculty of Health Sciences, School of Veterinary Medicine, Aristotle University of Thessaloniki, 54124 Thessaloniki, Greece; nikpap@vet.auth.gr; 6 Laboratory of Virology, Faculty of Veterinary Medicine, Ghent University, 9820 Merelbeke, Belgium; hans.nauwynck@ugent.be

**Keywords:** neuropathogenesis, pigs, maxillary nerve

## Abstract

Pseudorabies virus (PRV) is the causative agent for Aujeszky’s disease, a disease that mainly affects pigs and incidentally other domestic and wild animals. While PRV is almost always fatal, causing neurological disease independently of the age in non-porcine species, the development of neurological manifestation in its host species, the pig, highly depends on the age. In this study, an attempt was made to investigate the effect of nerve development on the outcome of virus infection and the effect of virus infection on the structure of nerves in piglets of various ages. For that reason, 42 pigs at the age of one (n = 14), three (n = 14) and five (n = 14) weeks were inoculated with 10^7^ TCID_50_ of PRV Kaplan strain and euthanized at one- or four-days post inoculation (DPI). The tissues of the trigeminal nervous pathway were collected and examined for virus replication (titration) in cell cultures for nerve morphology by light and transmission electron microscopy, and for viral antigen visualization by immunohistochemistry. The results showed that as the age of the pig increases, virus titers and clinical manifestations reduced, while, at the same time, myelin and axon development ceased. Following infection, the nerve structure was disrupted at all ages examined, being more prominent in one-week-old pigs compared to five-week-old pigs. In conclusion, the age-dependent PRV neuroinvasion in pigs seems to correlate with the morphological changes of neurons.

## 1. Introduction

*Suid herpesvirus 1*, also known as Aujeszky’s disease virus or pseudorabies virus (PRV), has been classified in the genus *Varicellovirus*, subfamily *Alphaherpesvirinae* and family *Herpesviridae* [1]. PRV is the causative agent for pseudorabies, a disease that mainly affects pigs and incidentally other domestic and wild animals. Although PRV was thought to infect only animals [2], recent data suggest that it is also possible to infect humans [3,4,5]. PRV has been eliminated from the domestic pigs of several countries in Europe and around the world due to the implementation of national eradication programs [6,7,8]. However, it is still endemic in domestic pigs of many other countries, while the fact that PRV circulates in the population of wild pigs worldwide represents a constant danger for PRV-free pig populations [9,10,11,12].

Infection of the pig with the highly neurotropic virus PRV is an excellent model for understanding the neuropathogenesis of alphaherpesviruses [13]. While PRV is almost always fatal, causing neurological disease independently of the age in non-porcine species, the severity of neurological manifestation in its host species, the pig, decreases as the age increases [14,15,16]. Neurological signs and death are the principal clinical manifestations prior to weaning, while the disease in older pigs is characterized by the development of respiratory symptoms in fatteners, abortion in sows and reversible infertility in boars. After primary replication in the nasopharyngeal mucosa, the virus spreads via nerves towards the central nervous system (CNS), where it causes non-suppurative meningoencephalitis. One of the main routes that PRV follows to the porcine CNS is the trigeminal nervous pathway that consists of three neuronal levels available for sequential virus invasion [17]. The neurons of the trigeminal ganglion represent the first neuronal level; the neurons of the pontine and spinal nucleus of the trigeminal nerve, which are located in the pons and the medulla oblongata, respectively, represent the second neuronal level; the neurons of thalamus and the Purkinje cells of the cerebellum represent the third neuronal level.

In this study, the interactions of PRV with nervous tissues were investigated in young pigs of different ages. As the maxillary nerve plays a pivotal role in the transmission of the virus from the periphery to the brain, virus invasion, spread and certain nerve characteristics measured at the level of this nerve (axon and myelin thickness) were compared in PRV infected and uninfected pigs of different ages [1,16,17].

## 2. Results

Piglets of all ages euthanized one DPI did not show any signs of the disease. On the other hand, pigs euthanized at four DPI showed clinical signs, the severity of which was dependent on their age: (i) the one-week-old piglets showed diminished appetite, nasal secretion, depression and unbalanced stepping, while they were continuously keeping their heads down sniffing at the floor; (ii) the three-week-old piglets showed less severe symptoms, e.g., diminished appetite and nasal secretion, while only two of them were depressed; (iii) the five-week-old pigs were in a good condition showing only a small decrease in their appetite.

**Figure 1:**
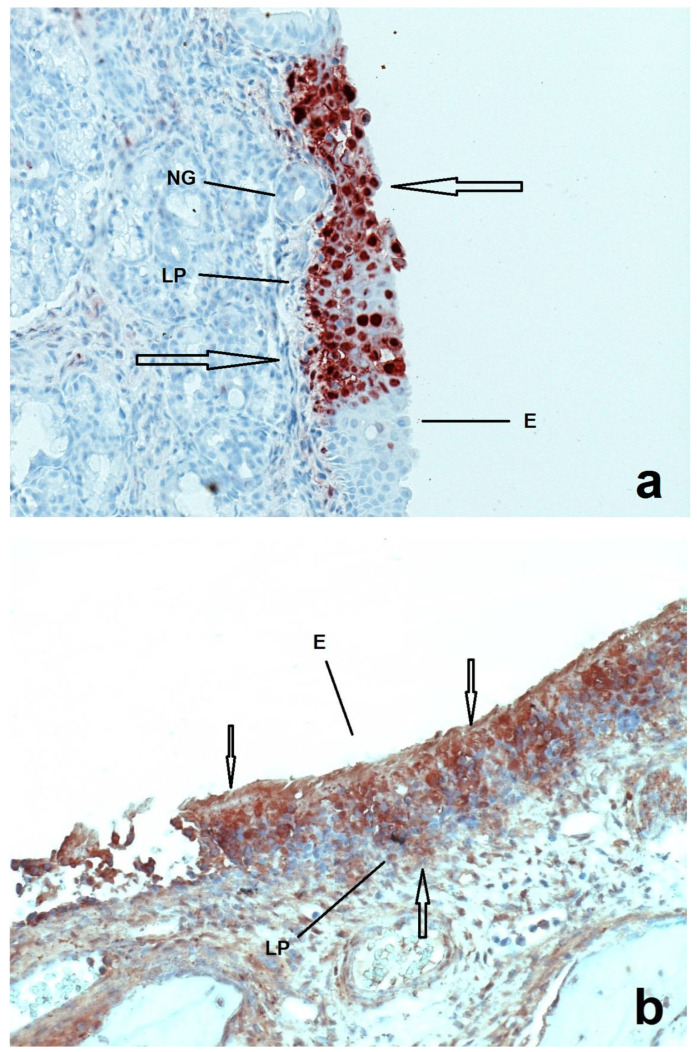
Sections of nasal mucosa of two pigs inoculated with Ka strain of PRV one week after their birth and euthanized at 1 (**a**) and 4 (**b**) DPI respectively. E = epithelium, LP = lamina propria, NG = nasal glands. At 1 DPI the infection (arrows) seems to be more localized in the epithelium compared to the 4 DPI, where the infection is more prominent.

Invasion and spread of PRV in the trigeminal nervous pathway of the infected pigs as demonstrated by virus titration is presented in Table 1. At both one and four DPI, Ka strain was present in the nasal mucosa of all age groups. At one DPI, the virus had been isolated only up to the trigeminal ganglion of the pigs of 1w- and 3w-groups, while no virus had been isolated in any neural tissue of the pigs of the 5w-group. At four DPI, the virus had been detected in all neuronal levels of the pigs of all age groups, with virus quantities being reduced with increasing age and neuronal level (Table 1). PRV antigens were detected in the nasal mucosa of all age groups of pigs at both one and four DPI, with virus spread being more extensive at four DPI (Figure 1).

**Figure 2:**
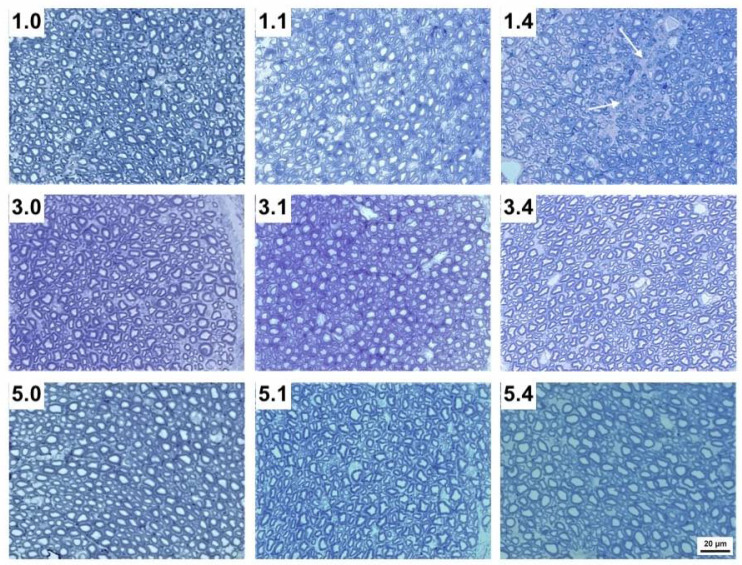
Transverse sections of the maxillary nerve of 1-week-, 3-week- and 5-week-old pigs without infection (1.0, 3.0 and 5.0, respectively), 1 DPI infection (1.1, 3.1 and 5.1, respectively) and 4 DPI infection (1.4, 3.4 and 5.4, respectively). Note the empty spaces (white arrows) within each nerve bundle after nerve disruption in 1.1 and particularly 1.4 pigs compared to 3.1 and 3.4, as well as to 5.1 and 5.4 pigs (Tolonidine blue staining photomicrographs).

**Figure 3:**
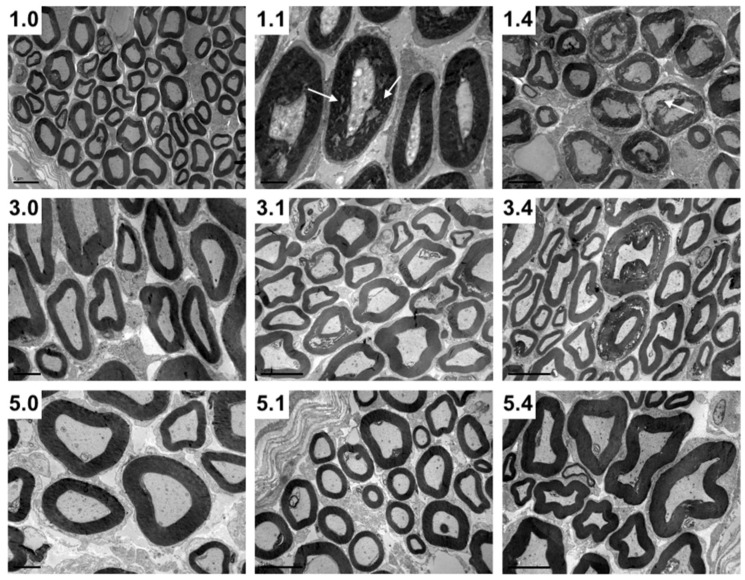
Transversal maxillary nerve sections of 1-week-, 3-week- and 5-week-old pigs without infection (1.0, 3.0 and 5.0, respectively), 1 DPI infection (1.1, 3.1 and 5.1, respectively) and 4 DPI infection (1.4, 3.4 and 5.4, respectively). Note the myelin disruption (white arrows) in 1.1 and particularly 1.4 pigs compared to 3.1 and 3.4, as well as to 5.1 and 5.4 pigs (Transmission electron microscopy photomicrographs).

As the maxillary nerve is the first neural tissue that comes into contact with PRV towards its entrance to the CNS, we further investigated its structure in uninfected and PRV-infected animals using the toluidine blue staining technique, which allows a more accurate visualization of the myelin shaft compared to IHC technique (Table 2). In uninfected pigs, axon diameter significantly increased up to three weeks of age, while myelin thickness increased up to five weeks of age (*p* < 0.05). Following infection, myelin thickness only increased by 12.8% at one DPI in one-week-old pigs (Table 2). At one DPI, infected axons showed a diameter increase in one- and in three-week-old pigs (24.4% and 5%, respectively), while at four DPI a slight decrease (8.8%) was observed in three-week-old pigs. By using light microscopy, a significant destruction in the nerve structure was clearly evident at one DPI and more noticeable at four DPI in one-week-old pigs compared to older pigs (Figure 2). Electron microscopy also showed that the myelin sheath was affected by the virus at all ages examined, being more prominent in one-week-old pigs compared to five-week-old pigs (Figure 3). More specifically, the myelin sheaths of one-week-old pigs showed areas of disruption with characteristic holes among myelin lamellae. In each age group, the degree of destruction was stronger at four DPI compared to one DPI. Therefore, among infected pigs the highest damage was seen in one-week-old pigs at four DPI, while no damage was detected in five-week-old pigs at one DPI (Figure 3). No virus particles were detected in any of the pigs.

## 3. Discussion

In a preliminary experiment using four pigs per age group, the PRV Ka strain at the dose of 10^7^ TCID_50_ killed 100% of one-week-old pigs by five–six DPI. It caused a slight depression followed by recovery in three-week-old pigs, while no obvious symptoms were observed in five-week-old pigs (data not shown). As specific immunity occurs five–seven days post-inoculation [18], we decided to follow virus infection at earlier time points of one and four DPI in the present study.

Neuropathogenesis of PRV is characterized by two directions of migration: (a) invasion, i.e., the depth or neuronal level that the virus can reach and (b) lateral spread, i.e., the degree of local replication or quantity of virus at a certain level. In this concept, the axon diameter is expected to mainly affect the movement of the virus towards a certain neuronal level (invasion), while the myelin sheath thickness is expected to affect the lateral spread of the virus. Eventually, the extent and combination of both may greatly determine the clinical outcome of infection [1,5,16,18]. In our study, PRV had equally invaded the first neuronal level of the trigeminal nervous pathway of the pigs of both one- and three-week-old groups as early as at one DPI (Table 1). However, further spread, as seen at four DPI, was most intense in 1w-group pigs compared to 3w-group pigs (Table 1). Invasion and spread of the 5w-group pigs were much more restricted. In a previous study [19], it was shown that myelination process continues during the postnatal life of the healthy pig. Moreover, it was shown that there is a drastic increase of myelin thickness and axon diameter of the pig during its first week of life (24.3% and 37.8%, respectively), which becomes less marked within the next couple of weeks (4.8% and 7.4%, respectively) and almost stabilizes until five weeks of age (4.6% and 0.2%, respectively). Apparently, this incomplete myelin and axon structures during the first week of life may be an explanation for the increased sensitivity of the piglets to PRV at that age. Papageorgiou and co-workers [19] have also shown by electron microscopy that as the age of the pigs increases, the myelin sheath becomes denser and more compact, a finding implying that PRV has to cross more layers in order to reach contact cells and therefore may show reduced neural spread.

Another finding in our study was the absence of infectious virus in maxillary nerve during the early stages of infection (one DPI) in the pigs of both 1w- and 3w-groups, while at the same time an infectious virus was present in the trigeminal ganglion (Table 1). This observation, also made in other studies [15], might be explained by the notion that naked virus is first transported towards the nucleus of the bipolar neuron in a non-infectious form, and after local replication, the virus moves anterogradely (away from nucleus) towards the periphery in an infectious form. The number of viral particles at these early stages is rather low, and this is possibly the reason why such particles were not detected by IHC or transmission electron microscopy. However, at a later stage at four DPI (when myelin damage is more advanced), the virus is able to cross the myelin sheath and infect surrounding cells.

The results of our study also suggest that probably early events during the interaction of PRV with axons of the maxillary nerve induce other structural changes in the axon and myelin sheath as early as one DPI (Figure 3), even before an infectious virus could be detected in the respective nerve of one- and three-week-old pigs. De Regge et al. [20] described a mechanism according to which the binding of virus glycoprotein gD alone in axons is able to trigger the formation of egress sites so that infectious virus can later be facilitated during its exit. A similar mode of action can also be valid for myelin, so that the binding of a viral component to a neuron may trigger myelin changes in advance before virulent virus is shed. As such changes were not detected in five-week-old pigs, they may be age dependent. Whether adjacent neurons can be infected through this cell-to-cell spread is not clear. According to Ohara et al. [21], PRV virions released from infected cells diffuse through the extracellular space outside the axons in the endoneurium, infecting non-neuronal cells. Could these non-neuronal cells be the intermediate link between two neighboring neurons? Although Schwann cells adjacent to infected neurons are often infected, the virus does not seem to spread from these non-neuronal cells [22]. Of course, it is necessary to mention that most of the studies with regard to PRV kinetics in neurons use mice or rats as animal models and not pigs, PRV’s natural host. According to Kritas et al. [16], viral neuropathogenesis in animal species which are not the natural host may be different, so extrapolation of findings during a PRV infection from non-porcine to porcine hosts may be misleading.

In conclusion, we found an association between the differentiation of axons and myelin sheaths of the maxillary nerve and the reduction of invasion and spread of PRV through this nerve during the first weeks of life in pigs. We speculate that the myelin sheath insulation is a key factor to protect pigs from neuronal viral spread and consequently from neurological disorders upon nasal PRV infection. The results of this study may contribute to scientific literature regarding neuropathogenesis of PRV and alphaherpesviruses, although further work is necessary to understand the underlying mechanisms.

## 4. Materials and Methods

### 4.1. Pig Inoculation and Collection of the Samples

Sixty-six (66) pigs from a conventional PRV-free farm in the province of Macedonia in the northern part of Greece were used. Blood samples from each piglet were examined by anti-PRV-gB ELISA (IDEXX Laboratories, Westbrook, ME, USA) prior to the start of the experiment to confirm the absence of antibodies against PRV.

The piglets were divided into three groups according to their age (Group 1w: 22 pigs—1 week old; Group 3w: 22 pigs—3 weeks old; Group 5w: 22 pigs—5 weeks old) (Table 3). From each group, 14 animals were intranasally inoculated with 1 mL inoculum containing 10^7^ TCID_50_ wild-type Ka strain, slowly administered by a catheter into both nostrils while the animal was placed on its back. The amount of virus was selected based on previous well documented protocols permitting clear observations of neurological manifestations [16]. For each group of infected animals another 8 uninfected piglets were used as negative controls (Table 3). Forty-two (42) pigs were sedated, anaesthetized and received transcardial perfusion of 4% paraformaldehyde solution in phosphate buffer saline (PBS; pH 7.4) in order to evaluate the effect of PRV on the morphometric parameters of the maxillary nerve. The remaining pigs were euthanized by electrocution and were used for virus isolation quantitation.

The pigs that received a transcardiac perfusion were first sedated by intramuscular injection of Azaperone (Stresnil 40 mg/mL, Vetoquinol, Lure, France) and subsequently anaesthetized by intravenous injection of Sodium Thiopental (Pentothal, Abbott Laboratories, Abbott Park, IL, USA) in heparinized saline (Heparin Sodium, LEO Pharma, Ballerup. Denmark). Exsanguination began by incision of the right auricle of the heart after opening the thoracic cavity. As a next step, perfusion was conducted by injecting saline solution from the left ventricle into the aorta in order to remove the blood from the circulatory system, followed by the fixative. All solutions were warmed to body temperature (37 °C). Subsequently, the nasal mucosa, maxillary nerve, trigeminal ganglion, pons, medulla oblongata, thalamus and cerebellum were extracted from each animal. The extraction of the maxillary nerve and the trigeminal ganglion from the pigs was based on the technique described by Meyerholz and Reznikov [23]. All the collected tissues were kept in 4% paraformaldehyde overnight at 4 °C. The maxillary nerves from the right part of the animals were used for the light and transmission electron microscopy. The tissues collected from the left part of the animals were dehydrated in ethanol, embedded in paraffin and cut in serial 3–5 μm vertical (or longitudinal for the maxillary nerve) sections on a microtome (HM315, Microm, Walldorf, Germany). The sections were cut at 5 (or 10 for the nasal mucosa and the trigeminal ganglion) different places equally distributed to ensure that the whole tissue was covered and were used for the immunohistochemistry technique. From the pigs euthanized by electrocution, the same tissues were collected and used for virus titration in swine testicle (ST) cell cultures (ATCC CRL-1746).

### 4.2. Immunohistochemistry (IHC)

After the deparaffinization in xylol and the following rehydration step in ethanol, the sections were placed in 3% H_2_O_2_ bath in PBS for 10 min to block endogenous peroxidase activity, washed in distilled water and PBS and subsequently incubated for 30 min at 37 °C with normal goat serum diluted 1/10 in PBS. Afterwards, the avidin-biotin-peroxidase (ABC) staining method was performed. Specifically, sections were incubated overnight at 4 °C with the polyclonal rabbit anti-PRV antibody, diluted 1/4000 in PBS. Sections were then washed in PBS and a biotinylated goat anti-rabbit antibody diluted 1/200 in PBS was added for 30 min at 37 °C. After washing in PBS, Avidin Biotin Complex (VECTASTAIN ABC Kit, Vector Laboratories, Burlingame, CA, USA) was placed on the sections and incubated for another 30 min at 37 °C. The visualization was conducted using AEC (Substrate chromogen ready to use, Dako Denmark A/S, Glostrup, Denmark) and subsequently the sections were counterstained with hematoxylin.

### 4.3. Antibodies and Virus

For producing rabbit polyclonal antibody against PRV, rabbits were injected with three doses of Geskypur PRV vaccine (Merial Animal Health, Woking, UK) 20 days apart. Twenty days after the last injection, rabbits were exsanguinated, and the collected blood serum was titrated by anti-PRV-gB ELISA and stored at −20 °C. The biotinylated goat anti-rabbit IgG antibody was purchased from Vector Laboratories (Burlingame, CA, USA). Goat normal serum was used as blocking serum.

For the inoculations we used the well-studied wild-type Kaplan (Ka) strain, which is virulent for newborn pigs (Kritas, 1994c). The Ka strain was kindly provided by the Laboratory of Virology, Faculty of Veterinary Medicine, University of Ghent.

### 4.4. Light and Transmission Electron Microscopy

The maxillary nerves from the right part of the animals were postfixed in 1% osmium tetroxide in phosphate buffer for 5 h (4 °C), dehydrated in ethanol and embedded in epoxy resin (Araldite CY 212, AGAR, Stansted Essex, UK). Semi-thin (1 μm) and ultra-thin (70–80 nm) cross sections were cut on an ultramicrotome (OM2, Reichert, Vienna, Austria). The semi-thin sections were stained with toluidine blue for sharpening of the histological images and observed under a light microscope (Axioplan, Zeiss, Oberkochen, Germany). The evaluation was conducted in the photomicrographs of at least ten optical fields (magnification ×40) that were randomly selected from 10 different nerve fiber bundles of the maxillary nerve adjusting the technique applied by Keuker et al. [24]. The photomicrographs were evaluated using an image analysis software (Image pro Plus, Media Cybernetics, Rockville, MD, USA). In addition, the ultra-thin sections were double stained with uranyl acetate and lead citrate, viewed with a JEOL JEM1011 (JEOL Ltd., Tokyo, Japan) transmission electron microscope at 80 kV and images were acquired with a Gatan ES500W camera (5794 W. Las Positas Blvd., Pleasanton, CA, USA).

### 4.5. Virus Quantitation

Either 20% or 10% (for nerves and trigeminal ganglion) suspensions in PBS were made from the collected tissues derived from the pigs euthanized by electrocution. The suspensions were homogenized by appropriate equipment (D160 Homogenizer, LabEx Ltd., EU) and they were centrifuged at 3000 rpm for 20 min at 4 °C. Subsequently, 0.1 mL (or 0.05 mL when the volume was small) of supernatant was inoculated on each of 4 wells of ST cells. The inoculated cell cultures were observed during 5 days for cytopathic effect. For the virus quantitation, 10-fold dilutions of the original suspensions were titrated on the cells and the titers were calculated by the Reed-Muench method.

### 4.6. Statistical Analysis

The obtained data were analyzed using SPSS 17.0 (IBM Corporation, Armonk, NY, USA). Specifically, one-way ANOVA was used for comparing the extracted means of the different age groups, while a Bonferroni post-hoc test was carried out in order to test the homogeneity of data. If the Levene index was not statistically significant, the results of one-way ANOVA were accepted. However, if the elements of the data were not normally distributed, inducing a statistically significant Levene index (<0.05), the results of one-way ANOVA were not accepted, and a non-parametric test (Kruskal–Wallis) was carried out. Thereafter, the non-parametric Mann–Whitney test was used for further analysis of the data, whenever that was necessary.

## Figures and Tables

**Table 1:** Invasion and spread of PRV (Ka strain) in the trigeminal nervous pathway of pigs of various ages.

			Virus Titer (log TCID_50_/g Tissue)—Mean Values					
Days Post Inoculation (DPI)	Age Group	Nr of Pigs	Nasal Mucosa	Maxillary Nerve	Trigeminal Ganglion	Pons- Medula Oblong	Cerebellum	Thalamus
1	Group 1w	3	7.2	0	1.25	0	0	0
	Group 3w	3	5.8	0	1.5	0	0	0
	Group 5w	3	5.4	0	0	0	0	0
4	Group 1w	3	6.3	3.5	4.7	4.3	3.4	2.75
	Group 3w	3	5.3	3.2	4.4	3.8	1.2	1.2
	Group 5w	3	5.3	1.7	2.8	3	1.3	0

w = week.

**Table 2:** Maxillary nerve morphology in non-infected and age-matched PRV-infected pigs.

	Infected Pigs				Uninfected Pigs			
Euthanisation Day	Age Group	Nr of Pigs	Myelin Thickness (μm)	Axon Diameter (μm)	Age Group	Nr of Pigs	Myelin Thickness (μm)	Axon Diameter (μm)
1 DPI	Group 1w	4	1.06 ^a^	3.01 ^c^	Group 1w	3	0.94 ^a^	2.42 ^a^
	Group 3w	4	1.00 ^b^	2.73 ^d^	Group 3w	3	0.98 ^b^	2.60 ^b^
	Group 5w	4	1.03 ^c^	2.65 ^b^	Group 5w	3	1.03 ^c^	2.61 ^b^
4 DPI	Group 1w	4	0.92 ^a^	2.40 ^a^	Group 1w	3	0.94 ^a^	2.42 ^a^
	Group 3w	4	0.99 ^b^	2.40 ^a^	Group 3w	3	0.98 ^b^	2.63 ^b^
	Group 5w	4	1.01 ^c^	2.63 ^b^	Group 5w	3	1.02 ^c^	2.66 ^b^

Different superscripts denote significantly different values regarding the data of each column (*p* < 0.05).

**Table 3:** Inoculation/euthanasia time and the related number of inoculated pigs and negative controls.

Type of Experiment	Age	Day of Inoculation	Day of Euthanization	Days Post Inoculation (DPI)	Number of Inoculated Pigs	Number of Negative Controls
Nerve Structure and Virus Inoculation	Group 1w	7	8	1	4	3
			11	4	4	3
	Group 3w	21	22	1	4	3
			25	4	4	3
	Group 5w	35	36	1	4	3
			39	4	4	3
Virus Titration	Group 1w	7	8	1	3	1
			11	4	3	1
	Group 3w	21	22	1	3	1
			25	4	3	1
	Group 5w	35	36	1	3	1
			39	4	3	1

## Data Availability

The data presented in this study are available in Supplementary Material.

## Supplementary Materials

The following supporting information can be downloaded at: https://www.mdpi.com/article/10.3390/pathogens11020157/s1, Table S1: Invasion and spread of PRV (Ka strain) in the trigeminal nervous pathway of pigs of various ages.

## References

[1] Mettenleiter T.C. (2000). Aujeszky’s disease (pseudorabies) virus: The virus and molecular pathogenesis—State of the art, June 1999. Vet. Res..

[2] Tischer B.K., Osterrieder N. (2010). Herpesviruses—A zoonotic threat?. Vet. Microbiol..

[3] Ai J.W., Weng S.S., Cheng Q., Cui P., Li Y.J., Wu H.L., Zhu Y.M., Xu B., Zhang W.H. (2018). Human Endophthalmitis Caused by Pseudorabies Virus Infection, China, 2017. Emerg. Infect. Dis..

[4] Yang H., Han H., Wang H., Cui Y., Liu H., Ding S. (2019). A Case of Human Viral Encephalitis Caused by Pseudorabies Virus Infection in China. Front. Neurol..

[5] Wong G., Lu J., Zhang W., Gao G.F. (2019). Pseudorabies virus: A neglected zoonotic pathogen in humans?. Emerg. Microbes Infect..

[6] Hahn E.C., Fadl-Alla B., Lichtensteiger C.A. (2010). Variation of Aujeszky’s disease viruses in wild swine in USA. Vet. Microbiol..

[7] MacDiarmid S.C. (2000). Aujeszky’s disease eradication in New Zealand. Aust. Vet. J..

[8] Muller T., Batza H.J., Schluter H., Conraths F.J., Mettenleiter T.C. (2003). Eradication of Aujeszky’s disease in Germany. J. Vet. Med. B Infect. Dis. Vet. Public Health.

[9] Meng X.J., Lindsay D.S., Sriranganathan N. (2009). Wild boars as sources for infectious diseases in livestock and humans. Philos. Trans. R. Soc. Lond. B Biol. Sci..

[10] Muller T., Conraths F.J., Hahn E.C. (2000). Pseudorabies virus infection (Aujeszky’s disease) in wild swine. Infect. Dis. Rev..

[11] Muller T., Hahn E.C., Tottewitz F., Kramer M., Klupp B.G., Mettenleiter T.C., Freuling C. (2011). Pseudorabies virus in wild swine: A global perspective. Arch. Virol..

[12] Ruiz-Fons F., Segales J., Gortzar C. (2008). A review of viral diseases of the European wild boar: Effects of population dynamics and reservoir role. Vet. J..

[13] Kramer T., Enquist L.W. (2013). Directional Spread of Alphaherpesviruses in the Nervous System. Viruses.

[14] Kritas S.K., Pensaert M.B., Mettenleiter T.C. (1994). Role of Envelope Glycoproteins gI, gp63 and gIII in the Invasion and Spread of Aujezky’s Disease virus in the Olfactory Nervous Pathway of the Pig. J. Gen. Virol..

[15] Kritas S.K., Pensaert M.B. (1994). Role of gp63 and gp III of Aujeszky’s Disease Virus in the Invasion of the Olfactory Nervous Pathway in Neonatal Pigs. Acta Vet. Hung..

[16] Kritas S. (1994). Neuropathogenesis of Wild-Type and Deleted Aujeszky’s Disease Virus Strains in Pigs with and without Specific Maternal Antibodies. Ph.D. Thesis.

[17] Kritas S.K., Pensaert M.B., Mettenleiter T.C. (1994). Invasion and spread of single glycoprotein deleted mutants of Aujeszky’s disease virus (ADV) in the trigeminal nervous pathway of pigs after intranasal inoculation. Vet. Microbiol..

[18] Mettenleiter T.C., Ehlers B., Muller T., Yoon K.-J., Teifke J.P. (2012). Herpesviruses. Diseases of Swine.

[19] Papageorgiou K.V., Grivas I., Chiotelli M., Panteris E., Papaioannou N., Nauwynck H., Kritas S.K. (2016). Myelin Sheath Development in the Maxillary Nerve of the Newborn Pig. Anat. Histol. Embryol..

[20] De Regge N., Nauwynck H.J., Geenen K., Krummenacher C., Cohen G.H., Eisenberg R.J., Mettenleiter T.C., Favoreel H.W. (2006). α-Herpesvirus glycoprotein D interaction with sensory neurons triggers formation of varicosities that serve as virus exit sites. J. Cell Biol..

[21] Ohara P.T., Tauscher A.N., LaVail J.H. (2001). Two paths for dissemination of herpes simplex virus from infected trigeminal ganglion to the murine cornea. Brain Res..

[22] Tomishima M.J., Enquist L.W. (2002). In vivo egress of an alphaherpesvirus from axons. J. Virol..

[23] Meyerholz D.K., Reznikov L.R. (2017). Simple and reproducible approaches for the collection of select porcine ganglia. J. Neurosci. Methods.

[24] Keuker J.I.H., Vollmann-Honsdorf G.S., Fuchs E. (2001). How to use the optical fractionator: An example based on the estimation of neurons in the hippocampal CA1 and CA3 regions of tree shrews. Brain Res. Protoc..

