# Supplementary material for: Age-Dependent Invasion of Pseudorabies Virus into Porcine Central Nervous System via Maxillary Nerve
Source: Pathogens. 2022 Jan 26;11(2):157. doi: 10.3390/pathogens11020157 (PMC8878659; doi:10.3390/pathogens11020157)
Supplement: Supplementary file 1 [file pathogens-11-00157-s001.zip › pathogens-1562901-supplementary.pdf]

Table S1. Invasion and spread of PRV (Ka strain) in the trigeminal nervous pathway of pigs of various ages

Virus Titre (log $TCID_{50}$/g tissue)

Days Post Inoculation (DPI)	Age group	Nr of pig	Nasal Mucosa	Maxillary nerve	Trigeminal ganglion	Pons - Medula Oblong.	Cerebellum	Thalamus
1	Group 1w	1	7.25	0	1.75	0	0	0
		2	6.75	0	2	0	0	0
		3	7.5	0	0	0	0	0
	**Mean (Group 1w)**		**7.2**	**0**	**1.25**	**0**	**0**	**0**
	Group 3w	4	5.75	0	1.75	0	0	0
		5	5.5	0	0	0	0	0
		6	6	0	2.75	0	0	0
	**Mean (Group 3w)**		**5.8**	**0**	**1.5**	**0**	**0**	**0**
	Group 5w	7	6	0	0	0	0	0
		8	5.2	0	0	0	0	0
		9	5	0	0	0	0	0
	**Mean (Group 5w)**		**5.4**	**0**	**0**	**0**	**0**	**0**
4	Group 1w	10	6.75	4	4.75	5	4.25	3.5
		11	5.5	2.9	4	3.5	2.5	2.5
		12	6.75	4	5.25	4.5	3.5	2.25
	**Mean (Group 1w)**		**6.3**	**3.5**	**4.7**	**4.3**	**3.4**	**2.75**
	Group 3w	13	4.25	3	4.75	4.25	1.75	1.75
		14	6	3.2	4.75	4	0	1.75
		15	5.75	3.3	3.75	3.25	1.75	0
	**Mean (Group 3w)**		**5.3**	**3.2**	**4.4**	**3.8**	**1.2**	**1.2**
	Group 5w	16	5.75	2.9	3	2.5	1.75	0
		17	4.25	2.2	2.25	3.25	0	0
		18	5.75	0	3.25	3.25	2	0
	**Mean (Group 5w)**		**5.3**	**1.7**	**2.8**	**3**	**1.3**	**0**

w=week
